# Pontine infarction with pure motor hemiparesis or hemiplegia: A prospective study

**DOI:** 10.1186/1471-2377-9-25

**Published:** 2009-06-15

**Authors:** Li Ling, Liangfu Zhu, Jinsheng Zeng, Songjie Liao, Suping Zhang, Jian Yu, Zhiyun Yang

**Affiliations:** 1 Department of Neurology and Stroke Center, the First Affiliated Hospital, Sun Yat-Sen University, No. 58 Zhongshan Road 2, Guangzhou, 510080, PR China; 2 Department of Neurology, Guangzhou Red Cross Hospital, the Fourth Affiliated Hospital, Jinan University, No.396 Tongfuzhong Road, Guangzhou, 510220, PR China; 3 Department of Neurology, the People's Hospital of Henan Province, No. 7 Wei Wu Road, Zhengzhou, 450003, PR China; 4 Department of Radiology, the First Affiliated Hospital, Sun Yat-Sen University, No. 58 Zhongshan Road 2, Guangzhou, 510080, PR China

## Abstract

**Background:**

The study aimed to prospectively observe the clinical and neuroimaging features of pontine infarction with pure motor hemiparesis (PMH) or hemiplegia at early stage.

**Methods:**

In 118 consecutive selected patients with the first-ever ischemic stroke within 6 hours after onset, fifty of them presented with PMH or hemiplegia and had negative acute computed tomography (CT) scans, then magnetic resonance imaging (MRI) confirmed the corresponding infarcts in pons or cerebrum. The clinical and neuroimaging features of the pontine infarctions were compared with those of cerebral infarctions.

**Results:**

The pontine infarction with PMH or hemiplegia accounted for 10.2% (12/118) of all first-ever ischemic stroke patients and 24% (12/50) of the patients with both PMH or hemiplegia and acute negative CT scans. Compared to the patients with cerebral infarction, the patients with pontine infarction had more frequency of diabetes mellitus (50.0% vs 5.3%, *P *= 0.001), nonvertiginous dizziness at onset (58.3% vs 21.1%, *P *= 0.036) and a progressive course (33.3% vs 2.6%, *P *= 0.011).

**Conclusion:**

The pontine infarction may present as PMH or hemiplegia with more frequency of nonvertiginous dizziness, a progressive course and diabetes mellitus. MRI can confirm the infarct location in the basal pons at early stage after stroke onset.

## Background

Pontine infarction is usually manifested by classical crossed syndromes such as Millard-Gubler syndrome, Foville syndrome, Raymond-Cestan syndrome [1]. These classical crossed pontine syndromes consist of ipsilateral peripheral cranial nerve palsies and contralateral movement disorders or sensory disturbances to the pontine lesions. However, some clinical observations have mentioned that pure motor hemiparesis (PMH) or hemiplegia can also be caused by pontine infarctions [2-8]. In 1965, Fisher [3] firstly described the lacunar syndrome of PMH associated with the pontine lacunar infarction. Later, Kim and colleagues [6] studied 37 patients with unilateral pontine base infarctions, and found 17 of them had PMH. Nighoghossian et al [8] reported pontine infarction represented 28.5% (6/21) in all patients with PMH in an 1-year study. The majority of pontine infarcts with PMH or hemiplegia are lacunar lesions, but some of them are larger than 20 mm [6,9]. Actually, in clinical practice, it is difficult to define the lesion location is in the pons or in the internal capsule-coronal radiate region only according to the early clinical manifestations of PMH or hemiplegia after stroke, especially in patients with negative brain computed tomography (CT) scans. Until now, the clinical and neuroimaging features, especially the details of magnetic resonance imaging (MRI) and magnetic resonance angiography (MRA) of pontine infarction patients with PMH or hemiplegia at early stage after stroke have not been well described. Moreover, the neurological deficits and prognosis have not been well investigated quantitatively. Therefore, in the present study, we prospectively observed the clinical and MRI(A) features, possible risk factors and prognosis of the patients of pontine infarction with PMH or hemiplegia, and compared them with those of capsule-coronal radiate region infarction who had similar clinical manifestations.

## Methods

### Patients

We prospectively selected consecutive patients with the first-ever ischemic stroke, who were admitted to our stroke center within 6 hours after symptom onset from May 1, 2002 to June 30, 2003. In all patients, after physical examination, the Oxfordshire Community Stroke Project (OCSP) classification [10] and the National Institutes of Health Stroke Scale (NIHSS) [11] were assessed on admission by the same neurologist. A brain CT scan was obtained immediately after the assessments. Then, MRI and MRA were performed within 72 hours after onset. According to the inclusive criteria, the patients who only had CT scans but couldn't perform MRI(A) because of their serious conditions were excluded from this study. Regular tests on admission consisted of blood cell count and urinalysis, fasting blood glucose level, electrolyte, transcranial Doppler, duplex sonography of the carotid and vertebral arteries, echocardiography, twelve-lead electrocardiography, etc.

The patients included in the study simultaneously fulfilled the following criteria: (1) A main clinical manifestation of PMH or hemiplegia. (2) An OCSP classification of lacunar infarction on admission. (3) An acute negative brain CT scan on admission. (4) An MRI performed within 72 hours after symptom onset showing a corresponding infarct in the pons or in the internal capsule-coronal radiate region. We described the early clinical and neuroimaging features of the pontine infarction patients presenting with PMH or hemiplegia, and compared the features, possible risk factors and prognosis of these patients with those of internal capsule-coronal radiate region infarction patients who had similar clinical manifestations.

The research protocol was approved by the local ethical committee for clinical research and all procedures involving the participant were conducted according to institutional guidelines in compliance with the regulations. Both oral and written informed consents were obtained from all participants.

### Clinical Assessments

All patients were assessed with the OCSP classification and the NIHSS on admission by the same neurologist. The neurological examinations were performed daily within the first week of stroke. Motor defects was graded in 0-V categories (0, no contraction; I, trace of contraction; II, active movement only with gravity eliminated; III, active movement against gravity; IV, active movement against resistance;V, noral strength) [12]. All patients were followed up 3 months after stroke. Barthel index (BI) [13] and modified rankin scale (mRS) [14] were used to evaluate quantitatively the degree of neurological functional recovery after stroke. "A progressive course" was defined as neurological worsening which is equal or greater than 1 point in the NIHSS for motor function within 3 days after admission [15].

### Brain CT and MRI Protocol

All patients performed brain CT scans (Toshiba X press/SX scanner, regular consecutive transverse plain scanning with sequences 10 mm slice thickness) immediately after addimission, and it was infarcts, but not others such as white matter lesions, that were regarded as the responsible lesions on CT scans. MRI was performed within 72 hours after onset (Siemens Magneton Vision 1.5 T scanner), including an axial T_1_-weighted spin-echo sequence (TR = 450 msec, TE = 14 msec, slice thickness = 7 mm, 0.7 mm spacing between slices) and an axial T_2_-weighted turbo- spin-echo sequence (TR = 4000 msec, TE = 120 msec, slice thickness = 7 mm, 0.7 mm spacing between slices), fluid-attenuated inversion recovery (FLAIR) sequence (TR = 9000 msec, TE = 110 msec, TI = 2500 ms) and MRA (three dimensional time-of flight technique sensitive to arterial flow, TR = 35 ms, TE = 7.2 ms). Diffusion-weighted images (TR = 5700 ms, TE = 139 ms, with gradual b values (0, 500, and 1000 s/mm, respectively, in the x, y, and z axes)) and apparent diffusion coefficient maps were performed additionally, which could distinguish new lesions from old ones. All CT and MR images were assessed by an experienced neuroradiologist who was blinded to the clinical symptoms and signs of the patients.

### Statistical Analysis

All statistical calculations were performed on microcomputer using SPSS13.0 (SPSS Inc., Chicago, IL, USA). Data are expressed as the mean ± SEM or median (range) for continuous variables and counts (percentage) for categorical variables. Comparisons between two groups were analyzed by the two-tailed Students' t-test or Mann-Whitney U test for continuous variables, and the two-tailed chi-square analysis or exact Fisher test for categorical variables. A *P *value less than 0.05 was considered statistically significant.

## Results

From May 1, 2002 to June 30, 2003, a total of 118 consecutive patients with the first-ever ischemic stroke were admitted to our stroke center within 6 hours after stroke onset. Among them, fifty patients fulfilled the inclusion criteria mentioned above, in which, 12 patients had pontine infarction and 38 patients had internal capsule-coronal radiate region infarction. MRI confirmed that the pontine infarction with PMH or hemiplegia accounted for 10.2% (12/118) of all first-ever ischemic stroke patients and 24% (12/50) of patients with PMH or hemiplegia who had acute negative brain CT.

The clinical features of patients with PMH or hemiplegia were compared between the pontine infarction patients and the internal capsule-coronal radiate region infarction patients (Table 1). None of the included 50 patients had hypoglycemia or orthostatic hypotension on admission. There were significant differences in the diabetes mellitus (50.0% vs 5.3%, *P *= 0.001), nonvertiginous dizziness at onset (58.3% vs 21.1%, *P *= 0.036) and a progressive course within 3 days after admission (33.3% vs 2.6%, *P *= 0.011) between the 2 groups.

**Table 1:** The Clinical Features of Pontine and Cerebral Infarction with PMH or Hemiplegia

	Patients with pontine infarctions (n = 12)	Patients with internal capsule-coronal radiate region infarctions (n = 38)	*P *value
Age (y, mean ± SD)	67.2 ± 8.3	68.1 ± 11.7	0.806
Female, n (%)	5 (41.7%)	16 (42.1%)	0.979
Hypertension, n (%)	8 (66.7%)	16 (42.1%)	0.138
Diabetes mellitus, n (%)	6 (50.0%)	2 (5.3%)	0.001
Coronary atherosclerotic heart disease, n (%)	1(8.3%)	2 (5.3%)	1.000
Nonvertiginous dizziness at onset, n (%)	7(58.3%)	8(21.1%)	0.036
Status at onset in quiet, n (%)	7(58.3%)	20(52.6%)	0.730
Progressing course of stroke, n (%)	4(33.3%)	1(2.6%)	0.011
Contralateral central facial palsy, n (%)	12(100.0%)	30(79.0%)	0.173
Contralateral central glossal palsy, n (%)	9(75.0%)	22(57.9%)	0.470
PMH, n (%)	7(58.3%)	14(36.8%)	0.188
Hemiplegia, n (%)	5(41.7%)	24(63.2%)	0.188
with contralateral Sensory dysfunction, n (%)	3(25.0%)	19(50.0%)	0.128
NIHSS on admission	6 (5, 12)	5(2, 11)	0.077

PMH, pure motor hemiparesis; NIHSS, the National Institutes of Health Stroke Scale.

**Figure 1:**
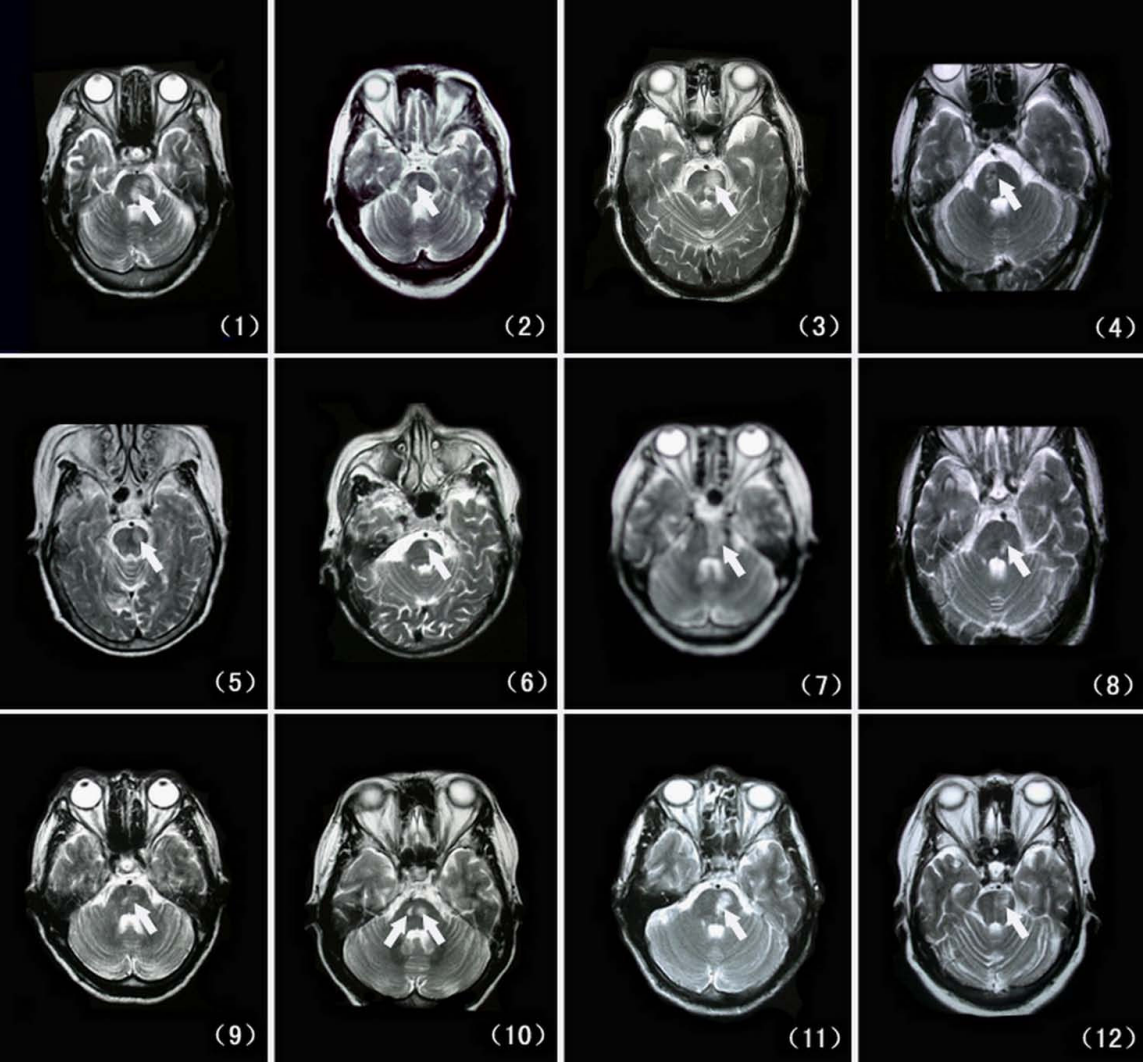
**Transversal T_2 _weighted imagings of magnetic resonance (MR) shows the infarction located in the dorsal surface of the pons, with the longitudinal strip or patch in shape (arrows show)**. The arabic numbers in brackets are patients' numbers.

**Figure 2:**
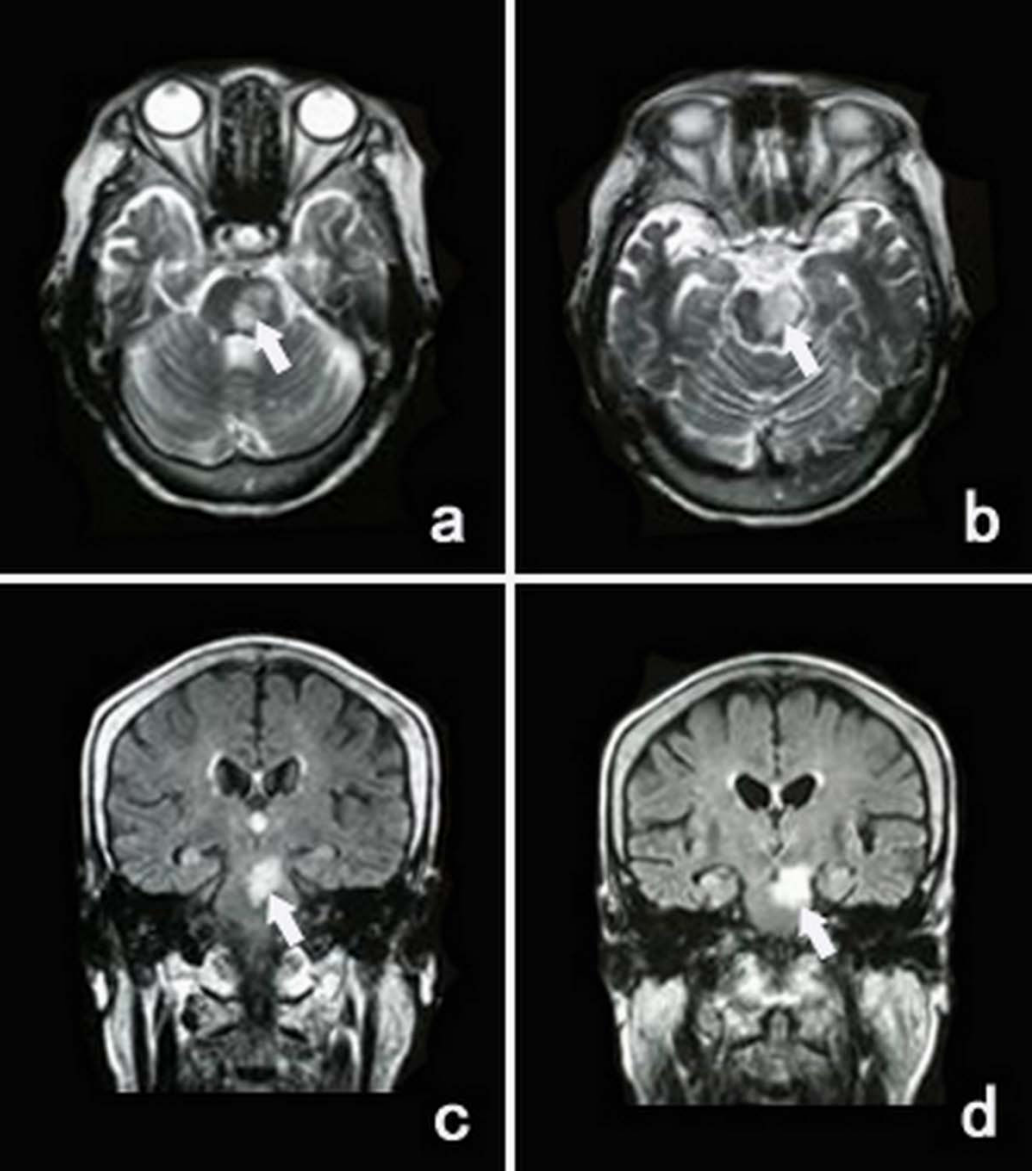
**Transversal T_2 _weighted imagings (a, b) and coronal FLAIR sequence (c, d) of MR show the infarction in the pons extending to the midbrain in case 1 (arrows show)**.

**Figure 3:**
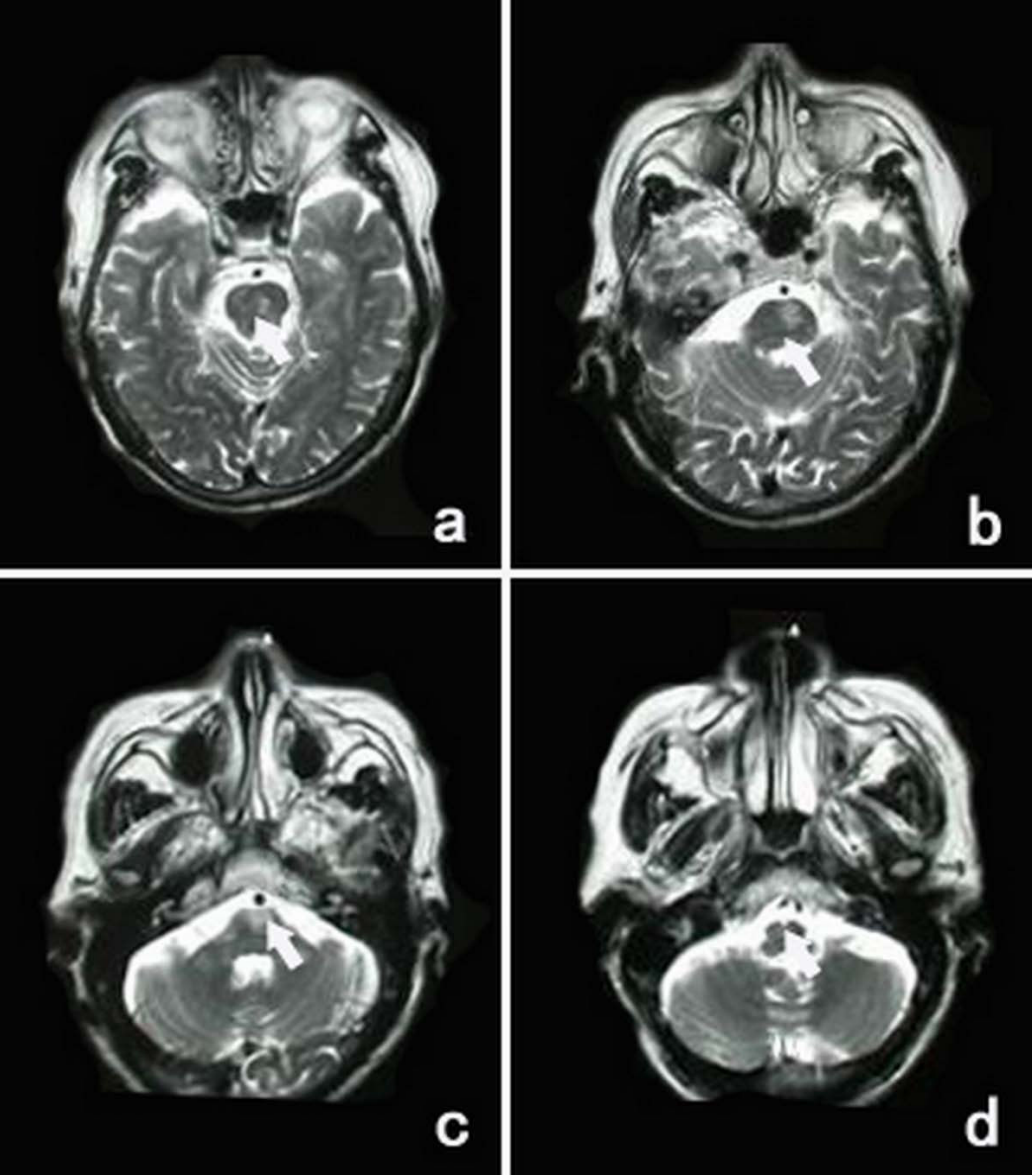
**Transversal T_2 _weighted imagings of MR (a-d) show the infarction in the pons expanding to the level of the pontomedullary junction in case 6 (arrows show)**.

Although early brain CT showed no lesion consistent with the main clinical signs (PMH or hemiplegia) in the 12 patients with pontine infarctions, MRI confirmed that an unilateral pontine infarction in 11 patients (8 patients in the left pons and 3 patients in the right pons) and bilateral pontine infarctions in only 1 patient (Figure 1, Table 2). All infarcts were located in the dorsal surface of the pons, with the longest diameter less than 15 mm in 4 patients, 15–20 mm in 6 patients, larger than 20 mm in 2 patients, and were longitudinal strip or patch in shape. MRA detected the atherosclerosis of intracranial arteries in 5 patients with pontine infarctions, and the large-vessel stenosis in 3 patients (Table 2). It was worth mentioning that although case 1 had pontine infarction extending to the midbrain (Figure 2), and case 6 had pontine infarction involving the level of the pontomedullary junction (Figure 3), both of them had no cranial nerve symptoms and signs consistent with the midbrain or medullary lesion.

**Table 2:** The MRI (A) Findings of Pontine Infarction Patients with PMH or Hemiplegia

Case No.	lesions on MRI	MRA findings
1	22 × 15 × 21 mm^3 ^in left basal and dorsal pontine pons and midbrain	Stenosis in bilateral PCA
2	12 × 10 × 12 mm^3 ^in right basal and dorsal pons	No intracranial large-vessel atherothrombosis or stenosis
3	21 × 18 × 14 mm^3 ^in left basal and dorsal pons, multiple lacunar infarctions in bilateral cerebral hemisphere and basal ganglia	Stenosis in left PCA and M_2 _segment of bilateral MCA
4	20 × 10 × 14 mm^3 ^in right basal and dorsal pon, multiple lacunar infarctions in bilateral parietofrontal lobe and basal ganglia	Atherosclerosis
5	15 × 12 × 20 mm^3 ^in left basal and dorsal pons and basal cerebral peduncle, multiple lacuner infarctions in bilateral subcortex regions and basal ganglia	Atherosclerosis
6	12 × 8 × 7 mm^3 ^in left basal and dorsal pons, lesion involving the level of the pontomedullary junction	Atherosclerosis
7	20 × 10 × 10 mm^3 ^in basal and dorsal part of left pons	No intracranial large-vessel atherothrombosis or stenosis
8	8 × 8 × 14 mm^3 ^in left basal and dorsal pons, multiple lacuner infarctions in bilateral parietofrontal lobes	No intracranial large- vessel atherothrombosis or stenosis
9	14 × 10 × 14 mm^3 ^in right basal and dorsal pons	Atherosclerosis
10	5 × 10 × 14 mm^3 ^in left basal and dorsal pons and 6 × 8 × 10 mm^3 ^in left basal and dorsal pons, multiple infarctions in bilateral subcortex regions and basal ganglia	Stenosis in M_2 _segment of lacunar right MCA and bilateral PCA
11	18 × 12 × 14 mm^3 ^in left basal and dorsal pons	No intracranial large-vessel atherothrombosis or stenosis
12	15 × 12 × 10 mm^3 ^in left basal and dorsal pons	Atherosclerosis

All the patients with PMH or hemiplegia were followed up to 3 months after onset, except 2 patients in the internal capsule-coronal radiate region infarction group were lost. In the pontine infarction group, only 1 patient had a recurrent infarction at contralateral basal ganglia. BI ranges from 40 to 100 (median, 100), in which 100 in 9 cases, 70 in 2 cases, and 40 in 1 case, and mRS ranges from 0 to 4 grade (median, 1), in which grade 0 in 5 cases, grade 1 in 4 cases, and grade 3 in 2 cases, and grade 4 in 1 case. In the internal capsule-coronal radiate region infarction group, one patient died. BI also ranges from 40 to 100 (median, 100) and mRS ranges from 0 to 6 grade (median, 1). Although "a progressive course" is more frequent in pontine infarction patients with PMH (Table 1), there was no significant difference in outcomes between the PMH patients with pontine infarction and those with internal capsule-corona radiata infarction (*P *> 0.05). Among 3 of 4 pontine infarction patients with progressive courses, one's BI was 40 while the others' were both 70, and mRS were 3, 3, 4 receptively, there was a significant difference in bad outcome (mRS is equal or greater than 3) between the the pontine infracted patients with and without a progressive course (3/4 vs 0/8, *P *= 0.018).

## Discussion

In our a-year study, the pontine infarction with PMH or hemiplegia accounted for 10.2% (12/118) in all first-ever ischemic stroke patients and 24% (12/50) in patients who presented with both PMH or hemiplegia and acute negative CT scans, and lacunar infarcts with PMH or hemiplegia accounted for 42% (50/118) of all first-ever ischemic stroke patients. Arboix and colleagues [16] reported that pure motor hemiparesis accounted for 12.7% of all first-ever acute stroke patients, while lacunar infarcts were found in 85% of the pure motor hemiparesis patients and accounted for 23% of all acute stroke patients. The main reason for more patients with lacunar infarction and PMH in our study is more lacunars in Asian population. Li and colleagues [17] reported that 468 patients (67.0%) were classified as lacunar infarct according to the OCSP in 669 consecutive patients with acute ischemic stroke in Hong Kong. The other study reported that lacunar infarction accounts for 62% in 205 consecutive ethnic South Asian ischemic stroke patients [18]. Another explanation for more patients with lacunar infarction and PMH in our study may be related to the way we selected the patients. Because the patients with first-ever ischemic stroke might undergo CT scan on admission and MRI(A) within 72 hours, some patients with too serious conditions to perform MRI(A) (e.g. malignant middle cerebral artery infarction or severe brain stem infarction) were excluded from this study. Our data suggest that the pontine infarction with PMH or hemiplegia is common in clinical practice. Although some previous studies have focused on the patients of pontine infarctions or posterior circulation infarctions and mentioned a few patients with PMH or hemiplegia among them, the clinical and neuroimaging features of pontine infarction with PMH or hemiplegia were not described in detail. The main findings of the previous studies are summarized in Table 3. In our study, all the 12 pontine infarction patients with PMH or hemiplegia had negative CT within 6 hours and positive MRI(A) within 72 hours after admission.

**Table 3:** Main Findings of Pontine Infarction Patients with PMH or Hemiplegia

Study	No. of patients	main clinical findings	risk factors	time and finding of CT scan	time and finding of MRI	MRA	outcome
Nighoghossian et al.[8] (1993)	6	PMH,	hypertension, gait ataxia, vertigo smoking, DM	36h(8–72h) after the stroke, CT(-)	20d(12–27d) after the stroke, MRI(+)	yes	the rate of disability is 86%
Kim et al. [6] (1995)	17	PMH, dysarthria, distinct lingual paresis	hypertension	not provided	not provided	two of them had occlusion of ICA	Not provided
Heo et al. (1996) [4]	1	PMH,	not provided	3d after stroke, CT(+)	1.5 month after stroke, MRI(+)	yes	Not provided
Kumral et al. [7] (2002)	39	PMH, dysarthria, ataxia	hypertension, hypercholesterolemia, DM, smoking	not provided	7d within the stroke	7d within the stroke	Good
Our study	12	PMH or hemiplegia, Nonvertiginous dizziness	DM	within 6 h after the stroke CT(-)	within 72 h after the stroke MRI(+)	within 72 h after the stroke	good

CT: computed tomographic; DM: diabetes mellitus, PMH: pure motor hemiparesis; MRI: magnetic resonance imaging; MRA: magnetic resonance angiography.

Until now, the exact risk factors and etiology for the pontine infarction are still unclear. Many previous studies reported that hypertension was the most important risk factor [2,5-7,9]. Other authors also found that diabetes was more frequently associated with lacunar infarctions. Arboix and colleagues' reports showed a increased occurrence of lacunar infarctions in diabetic patients, accompanied with a good prognosis [19]. In the present study, we found that diabetes mellitus is more frequent in pontine infarction patients with PMH or hemiplegia. As we know, like hypertension, diabetes mellitus can cause the injuries of small arteries and arterioles [20], especially in the retina, kidney and brain (mainly in thalamus, internal capsule and pons) [21]. Beside the penetrating arteries occlusion of paramedian or circumferential branches [6], some previous study also reported the high frequency of severe intracranial large-artery disease in posterior circulation infarctions including pontine infarctions [2,7,22,23]. A published study of 150 patients with acute isolated pontine infarctions showed that basilar artery branch disease (stenosis or occlusion, 39%) was the most etiology, followed by small-artery disease (21%) and cardioembolism (18%) [7]. Chan and Silver [24] reported a patient of right ventral pontine infarction was due to basilar artery stenosis. Bassetti et al [2] also thought basilar artery branch disease was the most common etiology of isolated pontine infarctions in their study. Taken together with our data of MRI and MRA, it is possible that occlusion of paramedian branches might be the main cause of pontine infarction with PMH or hemiplegia, but intracranial large-artery disease can not be excluded.

In the present study, the clinical manifestations were very similar in both the patients with pontine infarctions and those with internal capsule-coronal radiate region infarctions. Therefore, it's difficult to distinguish them only depending on the clinical signs of PMH or hemiplegia at early stage of stroke, especially the patients who had no visible lesion on brain CT. Actually, it has been reported that even lacunar infarction is poorly predictable according to PMH within 12 hours of the stroke event [25]. In this study, similar to diabetes mellitus, nonvertiginous dizziness at onset and a progressive course within 3 days after admission were also more frequently observed in the patients of pontine infarction with PMH or hemiplegia. It has been reported that diabetes mellitus is associated with a progressive course in ischemic stroke, increase infarct size and worsen stroke outcomes [22]. However, there may be no obvious relationship between diabetes mellitus and nonvertiginous dizziness in this study, because none of our patients had orthostatic hypotension on admission, and the nonvertiginous dizziness here occurring at onset was different from the postural dizziness caused by autonomic neuropathy of diabetes mellitus, the later is often accompanied with orthostatic hypotension and may last for a long time unless it was treated specially [26]. Therefore, the clinical features of pontine infarctions with PMH or hemiplegia might be associated with nonvertiginous dizziness at onset, a progressive course within 3 days after admission and the history of diabetes mellitus.

As we know, MRI is more sensitive and specific than CT to identify the infarcts location of ischemic stroke at early stage after onset, especially to detect small or pontine lesions [2,5,7,8,27]. Moulin and colleagues studied 100 patients with ataxic hemiparesis after first stroke, and found that the most common location of lesions were the internal capsule (39%), followed by the pons (19%). There were 14% of their patients had no visible lesion on brain CT, and MRI was performed in 23 patients and confirmed the location of the lesion in half of CT negatives [27]. In our study, acute brain CT was not able to find any lesion consistent with the main clinical signs in the 12 patients of pontine infarctions with PMH or hemiplegia, but MRI confirmed that the diameter for the majority of the pontine infarctions was smaller than 20 mm, and all of infarcts located in the dorsal surface of the pons, an anatomic area in which the pyramidal tract passes through, but preserving protuberance calote in which the nuclei of the cranial pairs are located. MRA showed intracranial arteries atherosclerosis in 5 patients and a large-vessel stenosis in 3 patients, which suggests MRA may provide more information for the possible etiology of pontine infarctions.

Most of the previous studies showed a good short-term prognosis in the patients with lacunar infarctions in pons [2,7,28]. In the present study, we observed a good short-term prognosis in the patients of pontine infarctions with PMH or hemiplegia in a 3-month follow-up. Although "a progressive course" is more frequent in pontine infarction patients with PMH, there was no significant difference in outcomes between the PMH patients with pontine infarction and those with internal capsule-corona radiata infarction. However, compared to those without a progressive course, the pontine infracted patients with a progressive course had a bad outcome. Further investigations are needed to evaluate the long-term prognosis in more patients of the pontine infarction with PMH or hemiplegia.

## Conclusion

This study suggests that the pontine infarction with PMH or hemiplegia is a common clinical situation, which is worth receiving more attention. This subtype of pontine infarction patients might manifest PMH or hemiplegia, with nonvertiginous dizziness, a progressive course and the history of diabetes mellitus being more frequently. Though no visible lesion on brain CT on admission, MRI can confirm the infarct location in the basal pons at early stage after stroke onset.

## Authors' contributions

LL acquired data, performed statistical analysis, analyzed and interpreted data, and wrote and critically revised the manuscript. LZ acquired data, performed statistical analysis, analyzed and interpreted data. JZ designed and the study, obtained funding, performed the statistical analysis, interpreted the results and critically revised the manuscript. SL, SZ and JY assisted with the data collection. ZY assessed the CT and MR images. All authors read and approved the final manuscript.

## Pre-publication history

The pre-publication history for this paper can be accessed here:


